# Supplementary material for: Primordial aqueous alteration recorded in water-soluble organic molecules from the carbonaceous asteroid (162173) Ryugu
Source: Nat Commun. 2024 Jul 10;15:5708. doi: 10.1038/s41467-024-49237-6 (PMC11237059; doi:10.1038/s41467-024-49237-6)
Supplement: Supplementary file 1 — Supplementary Information [file 41467_2024_49237_MOESM1_ESM.pdf]

Supplementary Information

Primordial aqueous alteration recorded in water-soluble organic molecules from the carbonaceous asteroid (162173) Ryugu

Yoshinori Takano[1,2*], Hiroshi Naraoka[3], Jason P. Dworkin[4], Toshiki Koga[1], Kazunori Sasaki[2,5], Hajime Sato[5], Yasuhiro Oba[6], Nanako O. Ogawa[1], Toshihiro Yoshimura[1], Kenji Hamase[7], Naohiko Ohkouchi[1], Eric T. Parker[4], José C. Aponte[4], Daniel P. Glavin[4], Yoshihiro Furukawa[8], Junken Aoki[9], Kuniyuki Kano[9], Shin-ichiro M. Nomura[10], Francois-Regis Orthous-Daunay[11], Philippe Schmitt-Kopplin[12,13,14], Hayabusa2-initial-analysis SOM team[#], Hisayoshi Yurimoto[15], Tomoki Nakamura[8], Takaaki Noguchi[16], Ryuji Okazaki[3], Hikaru Yabuta[17], Kanako Sakamoto[18], Toru Yada[18], Masahiro Nishimura[18], Aiko Nakato[18], Akiko Miyazaki[18], Kasumi Yogata[18], Masanao Abe[18], Tatsuaki Okada[18], Tomohiro Usui[18], Makoto Yoshikawa[18], Takanao Saiki[18], Satoshi Tanaka[18], Fuyuto Terui[19], Satoru Nakazawa[18], Sei-ichiro Watanabe[20], Yuichi Tsuda[18], and Shogo Tachibana[18, 21]

[1] Biogeochemistry Research Center (BGC), Japan Agency for Marine-Earth Science and Technology (JAMSTEC), Natsushima, Yokosuka 237-0061, Japan.

[2] Institute for Advanced Biosciences (IAB), Keio University, Kakuganji, Tsuruoka, Yamagata 997-0052, Japan.

[3] Department of Earth and Planetary Sciences, Kyushu University, 744 Motooka, Nishi-ku, Fukuoka 819-0395, Japan.

[4] Solar System Exploration Division, NASA Goddard Space Flight Center, Greenbelt, MD 20771, USA.

[5] Human Metabolome Technologies Inc., Kakuganji, Tsuruoka, Yamagata 997-0052, Japan.

[6] Institute of Low Temperature Science (ILTS), Hokkaido University, N19W8 Kita-ku, Sapporo 060-0819, Japan.

[7] Graduate School of Pharmaceutical Sciences, Kyushu University, Fukuoka 812-0054, Japan.

[8] Department of Earth Material Science, Tohoku University, Sendai 980-8578, Japan.

[9] Department of Health Chemistry, Graduate School of Pharmaceutical Sciences, The University of Tokyo, Hongo, Tokyo 113-0033, Japan.

[10] Department of Robotics Graduate school of Engineering, Tohoku University, Sendai, 980-8579, Japan.

[11] Université Grenoble Alpes, Centre National de la Recherche Scientifique (CNRS), Centre National d'Etudes Spatiales, L'Institut de Planétologie et d'Astrophysique de Grenoble, 38000 Grenoble, France.

[12] Technische Universität München, Analytische Lebensmittel Chemie, 85354 Freising, Germany.

[13] Max Planck Institute for Extraterrestrial Physics, 85748 Garching bei München, Germany.

[14] Center for Research and Exploration in Space Science and Technology, NASA Goddard Space Flight

Center, Greenbelt, MD 20771, USA.

[15] Department of Earth and Planetary Sciences, Hokkaido University, Sapporo 060-0810, Japan.

[16] Department of Earth and Planetary Sciences, Kyoto University, Kyoto 606-8502, Japan.

[17] Department of Earth and Planetary Sciences, Hiroshima University, Higashi-Hiroshima 739-8526, Japan.

[18] Institute of Space and Astronautical Science (ISAS), Japan Aerospace Exploration Agency (JAXA), Sagamihara 252-5210, Japan.

[19] Kanagawa Institute of Technology, Atsugi 243-0292, Japan.

[20] Department of Earth and Environment Sciences, Nagoya University, Nagoya 464-8601, Japan.

[21] UTokyo Organization for Planetary and Space Science (UTOPS), University of Tokyo, 7-3-1 Hongo, Tokyo 113-0033, Japan.

***Corresponding author:**

E-mail: takano (a) jamstec.go.jp

#The Hayabusa2-initial-analysis SOM (Soluble Organic Matter) team:

Hiroshi Naraoka, Yoshinori Takano, Jason P. Dworkin, Kenji Hamase, Aogu Furusho, Minako Hashiguchi, Kazuhiko Fukushima, Dan Aoki, José C. Aponte, Eric T. Parker, Daniel P. Glavin, Hannah L. McLain, Jamie E. Elsila, Heather V. Graham, John M. Eiler, Philippe Schmitt-Kopplin, Norbert Hertkorn, Alexander Ruf, Francois-Regis Orthous-Daunay, Cédric Wolters, Junko Isa, Véronique Vuitton, Roland Thissen, Nanako O. Ogawa, Saburo Sakai, Toshihiro Yoshimura, Toshiki Koga, Haruna Sugahara, Naohiko Ohkouchi, Hajime Mita, Yoshihiro Furukawa, Yasuhiro Oba, Yoshito Chikaraishi, Takaaki Yoshikawa, Satoru Tanaka, Mayu Morita, Morihiko Onose, Daisuke Araoka, Fumie Kabashima, Kosuke Fujishima, Hajime Sato, Kazunori Sasaki, Kuniyuki Kano, Shin-ichiro M. Nomura, Junken Aoki, Tomoya Yamazaki, Yuki Kimura.

Supplementary Notes

Sample process used for the initial analysis of Ryugu chamber A and C samples

A summarized description of the onsite process by which samples were collected from the asteroid Ryugu was reported by the Hayabusa2 International Team [1-3]. This description was followed by the initial analysis team [4-9] with regard to 1) chemistry (i.e., comprehensive description), 2) petrology and mineralogy for coarse grains (i.e., mm-sized grains), 3) petrology and mineralogy for fine grains (i.e., <100 μm-sized grains), 4) volatile components (i.e., noble gas and inorganic and organic gases), 5) insoluble organic matter (IOM) (i.e., macromolecular organic matter), and 6) soluble organic matter (SOM) (i.e., extractable organic molecules), in combination with the phase 2 curational description [10-12]. A photograph of the samples (A0106 and C0107) before extraction is shown in Figure S1. We present a concept based on the dimensions of chemical resolution and chemical variation (Figure S2). Further science proposals after the initial analysis will be advanced by the international announcement of opportunity (AO) in the Astromaterials Science Research Group (ASRG) of ISAS/JAXA.

Supplementary Methods

Sequential solvent extraction for Ryugu A0106 and C0107

The detailed procedure for extracting organic molecules from the Ryugu samples has been described in the literature [8] and the scheme (Figure S3). In brief, we extracted organic molecules from the A0106 (13.08 mg) and C0107 (10.73 mg) Ryugu samples with hot water (TAMAPURE-AA, Tama Chemicals Co., Ltd.) at 105 °C for 20 h in N_2-purged and flame-sealed glass ampoules. After the extraction, the contents were transferred from the ampoule to a glass vial. The glass vial was centrifuged for 8 min at 14,000 rpm, after which the supernatant was transferred to another sample extraction vial. The glass ampoule was rinsed with 200 μL of H_2O, and then the H_2O solution was transferred to the residue-containing vial, which was further mixed by shaking. The glass vial was centrifuged for 8 min at 14,000 rpm, after which the supernatant was transferred to the sample extraction vial. This step was repeated, and then the combined 600 μL of solution was mixed well by shaking (i.e., the water/rock ratio was normalized to 600 μL of H_2O:10 mg of initial bulk). The hot water extract was split into 250 μL (KU) and 250 μL (GSFC) for amino acid analysis and 100 μL (JAMSTEC) for the analysis in the present report and that of [13].

Separate aggregates of A0106 (17.15 mg) and C0107 (17.36 mg) were extracted by a series of organic solvents with the assistance of ultrasonication (15 min) in the following order: hexane (hexane 5000, FUJIFILM Wako, 200 μL × 3), dichloromethane (DCM, dichloromethane 5000, FUJIFILM Wako, 200 μL × 3), methanol (MeOH, QTOFMS grade, FUJIFILM Wako, 200 μL × 3) and H_2O (200 μL × 3, TAMAPURE-AA, Tama Chemicals Co., Ltd.). After sonication and centrifugation (12,000 rpm, 5 min), the liquid extract was decanted. For further details, please refer to the reference [8]. We concurrently performed the same

procedure with the Murchison meteorite and the Murray meteorite [8,14,15] to assess CM group references with the same normalization of the water/rock ratio (i.e., 600 µL of H_2O: 10 mg of initial bulk each). The hot water extract was also analyzed using the same procedure as in the present report. Prior to analysis, all glassware used was heated in an oven at 500 °C for 3 h to remove possible organic contaminants.

Supplementary Discussion

Diverse and unique colors of the sequential solvent extracts

The colors of the sequential solvent extracts are diverse and chemically interesting, as shown in Figure S5. We observed that the hot water extracts (#7-1) from Ryugu A0106 and C0107 were colorless and transparent after extraction. Then, we observed diverse colored supernatants during sequential extractions from the aggregate samples. The colors of the extracts indicate the chemical features of the extractable organic molecules, which inherent the affinity of the solvent from low to high polarity (i.e., they depend on hydrophilicity, hydrophobicity, and amphiphilicity [8,16]. Potentially, dissolved inorganic species and organic–inorganic complex molecules exhibit chromaticity depending on the solvent affinity [13,16]. Figure S6 represents the soluble nitrogen from the initial bulk to the sequential extraction, i.e., the fraction of SOM (f_{SOM}) normalized by the nitrogen-based relative abundance (%) for Ryugu A0106, C0107 and the CI reference of Orugeil. Notably, the extractable inorganic carbon and inorganic nitrogen balance are operationally included in the inorganic fraction ($f_{Inorganics}$) and soluble organic fraction (f_{SOM}). Here, the theoretical mass balance equation of the carbon and nitrogen isotopic compositions is expressed as follows:

$$f_{IB} \times \delta^{13}C_{IB} = f_{Inorganics} \times \delta^{13}C_{Inorganics} + f_{SOM} \times \delta^{13}C_{SOM} + f_{IOM} \times \delta^{13}C_{IOM} \quad [i]$$

$$f_{IB} \times \delta^{15}N_{IB} = f_{Inorganics} \times \delta^{15}N_{Inorganics} + f_{SOM} \times \delta^{15}N_{SOM} + f_{IOM} \times \delta^{15}N_{IOM} \quad [ii]$$

where f is the number of moles of carbon and nitrogen as,

$$f_{IB} = f_{Inorganics} + f_{SOM} + f_{IOM} \quad [iii]$$

Since this formula is a theoretical equation, we note that inorganic carbon and nitrogen are practically combined with f_{SOM} during wet chemical processes [13], and volatile carbon and nitrogen are not included in f_{IOM}. To date, organic analysis of the pristine initial bulk (#1), hexane (#2), dichloromethane (DCM) (#3), methanol (MeOH) (#4), and water (#5) fractions for the sequence from low polarity to high polarity and hot water (#7-1, this study; #7-2 HCl), DCM/MeOH (#8), formic acid (FA) (#9, this study), hydrochloric acid (HCl) (#10, this study), and IOM (#11) fractions has been classified within the scope of soluble organic molecules [13, 17-21]. We obtained ultraviolet (UV)/visible absorption spectra of the extracts to acquire further organic chemical information and search for novel organic molecules [22,23].

UV–vis spectroscopy of the organic extracts

The UV–vis spectra of all #5 extracts (H_2O extracts) show no clear peak, although the spectra of the extracts of Ryugu A0106 and C0107 have a trace peak at ~204 nm (Figure S7 A–D). The spectra of the Ryugu #7-1 extracts (hot H_2O extracts) showed a peak at ~214 nm. A slight peak at ~214 nm was present in the spectrum of the Orgueil extract but was absent in that of the serpentine blank extract (Figure S7 E–H). The origin of this signal is unclear, but many organic compounds, including N-containing organic compounds, have a peak in this range [24]. The spectra of the Ryugu #7-2 extracts (HCl extracts) were similar to those of serpentine (Figure S7 I–L). The spectrum of the Orgueil extract clearly differs from that of serpentine and Ryugu, showing two peaks at ~225 and ~338 nm. The two characteristic peaks in the HCl extract do not appear in the reference HCl solution but can be attributed to $FeCl_3$ in HCl. Thus, the spectrum indicates that this extract contains Fe^{3+}. The absorption of short-wavelength visible light would also be consistent with the visible yellowish color of this extract. All #9 extracts (formic acid extracts) showed a peak at ~207 nm, which corresponded to a signal from formic acid (Figure S7 M–P). However, N-containing organic compounds also have a peak in this range [24], and N-heterocyclic compounds were found in the #9 extracts of Ryugu samples and the Orgueil meteorite by SALDI-MS (Figure S8). Thus, the compounds may provide a minor contribution to this signal. The extracts of Ryugu samples and the Orgueil meteorite additionally showed a peak at ~282 nm. This signal is attributed to Fe^{3+}, as shown by the spectrum of the HCl solution containing $FeCl_3$ (Figure S7).

High-resolution mass spectrometry of the pinkish formic acid extracts

We traced the molecular characterization of the pinkish formic acid extracts (#9) and the nitrogen isotopic profiles of the “extract” during the sequential extraction until the final insoluble organic matter (IOM) was obtained (Figure S8). The extracted formic acid fraction (#9) was dropped in a pit on a glass slide and dried on a clean bench at ambient temperature. A platinum sputtering deposit was applied to the glass slide for surface-assisted laser desorption/ionization (SALDI) mass spectrometry (MS) [25] [73]. Each spectrum contains many unassigned mass signals, but the elemental formula of $C_nH_{2n-m}N$ (n = 10~23, m = 6~10) is exclusively contained in the #9 extracts of Ryugu samples and the Orgueil meteorite (see also Figure S8). These compounds have been found in carbonaceous chondrites and are assigned as N-heterocyclic compounds, including alkylpyridines [8,17,26]. The contents were higher in C0107 than in A0106 but lower than those in the Orgueil and Murchison meteorites. The relative abundances between differently saturated series (e.g., $C_nH_{2n-6}N$ and $C_nH_{2n-8}N$) indicate that the Ryugu samples and the Orgueil meteorite are comparable, while the Murchison meteorite is enriched in a more saturated formula. A difference in the saturation of CHN compounds was previously found between less saturated CHN compounds in CM and more saturated CHN compounds in CR chondrites [14]. The difference between the Ryugu/Orgueil and Murchison meteorites implies that hydrogenation was less substantial in Ryugu and the parent asteroid of the Orgueil meteorite. Carbon number

distributions in each series (e.g., n = 17~21 in $C_nH_{2n-8}N$ of Ryugu C0107 and n = 10~19 in $C_nH_{2n-8}N$ of the Murchison extract [8]) show that the Ryugu samples and Orgueil meteorite contain larger CHN compounds than the Murchison meteorite, suggesting that the elongation reaction of the carbon chain was more substantial in their parent asteroids. This result may be consistent with more substantial aqueous reactions in Ryugu and the parent asteroids of CI chondrites than in the Murchison CM chondrite [4]. The carbon number distributions also show that the CHN compounds in A0106 are slightly smaller than those in C0107, implying a potential difference in the chain elongation reaction in the asteroid Ryugu.

Spectroscopic FTIR references for the soluble components

To further confirm the presence of soluble organic matter, FTIR spectra of the DCM and methanol extracts from the Ryugu samples were obtained, as shown in Figure S9 (A0106 and C0107). The predominant absorption at ~1000 cm^{-1} is derived from the Si-O stretching band of silicates in both extracts. Differences in the width and shape of the Si-O band between the two samples might be attributable to differences in the composition, size, and/or shape of the silicate grains [27]. In addition, the A0106 extract shows several absorptions at 1300-1600 cm^{-1}, which might be attributed to the aromatic C=C and/or N-heterocyclic C=N bands [28]. According to the distribution of alkylated N-heterocycles in the A0106 extract [8], the observed absorptions, as well as the weak absorptions at ~2900 cm^{-1} (C-H stretch), might be partially derived from N-heterocycles. In contrast, the C=C/C=N and C-H stretching bands were insignificant for the C0107 extract. A similar signature is also inferred from the distribution of N-heterocycles, in which the degree of alkylation in C0107 is less than that in A0106 (Figure S9). These features may be explained by the different histories of both samples on the parent body. Since the A0106 sample is derived from the surface of the asteroid Ryugu, it should have been exposed to energetic particles, such as cosmic rays and solar wind, which may modify organics and minerals [29]. The A0106 sample shows a tiny absorption band at ~3600 cm^{-1} attributed to OH stretching in minerals [30], which could be caused by residual water on the parent body.

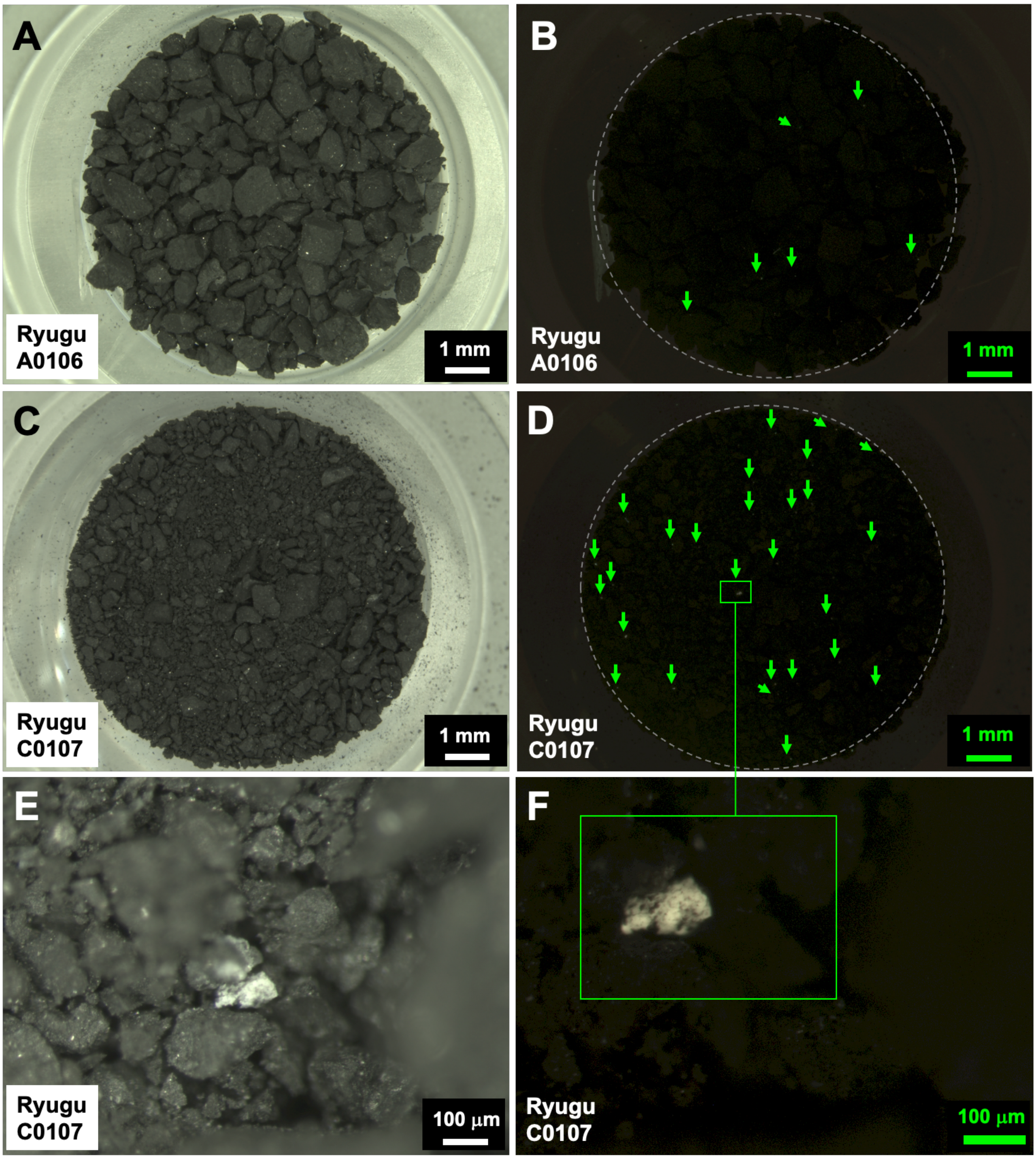

Supplementary Figure S1. Profiles of samples obtained from the asteroid Ryugu (162173) in this study. **(A, B)** Photographs of initial sample A0106 (a total of 38.4 mg, with normal light and UV light under a microscope) obtained from the asteroid Ryugu (162173) at the 1st touchdown sampling location [1,2]. **(C, D, E, F)** Photographs of initial C0107 samples (37.5 mg in total, with normal light and UV light under a microscope) obtained from the asteroid Ryugu (162173) at the 2nd touchdown sampling location [1,2]. Here, we observed carbonate fluorescence (indicated by green arrows) under UV light under the microscope of Leica M205 FA microscope.

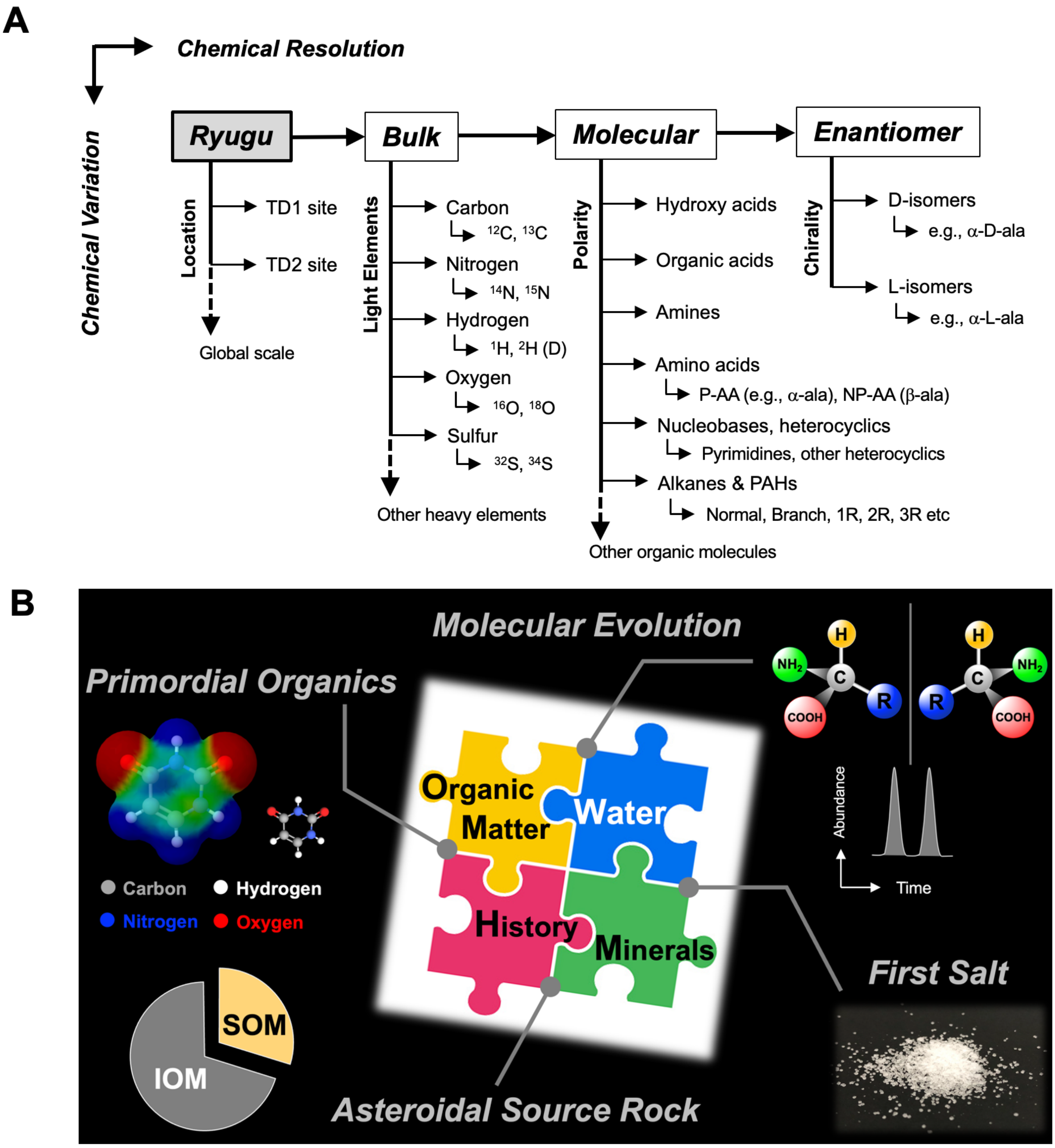

Supplementary Figure S2. Exploring the asteroid Ryugu by organic astrochemical approaches.
(A) Soluble organic surveys for chemical resolution and variation in the asteroid Ryugu. The locations for surface and subsurface sampling are important anchor points. The bulk profiles for light elements of CNHOS [8,31], molecular profiles within the wide polarity range for hydrophilic, semi-hydrophilic and hydrophobic fractions [8,13,18-21], and enantiomer profiles for symmetric amino acid isomers [8,18,19] were systematically classified for target analysis. It is important to merge the primary description with spatial distribution [32], molecular growth features [33] and a comprehensive molecular atlas [34]. **(B)** Conceptual diagram of the achievements in the analysis of soluble organic molecules in Ryugu samples. The aqueous alteration was determined by the latest organic chemical description for Ryugu ([8,13,31] and this study).

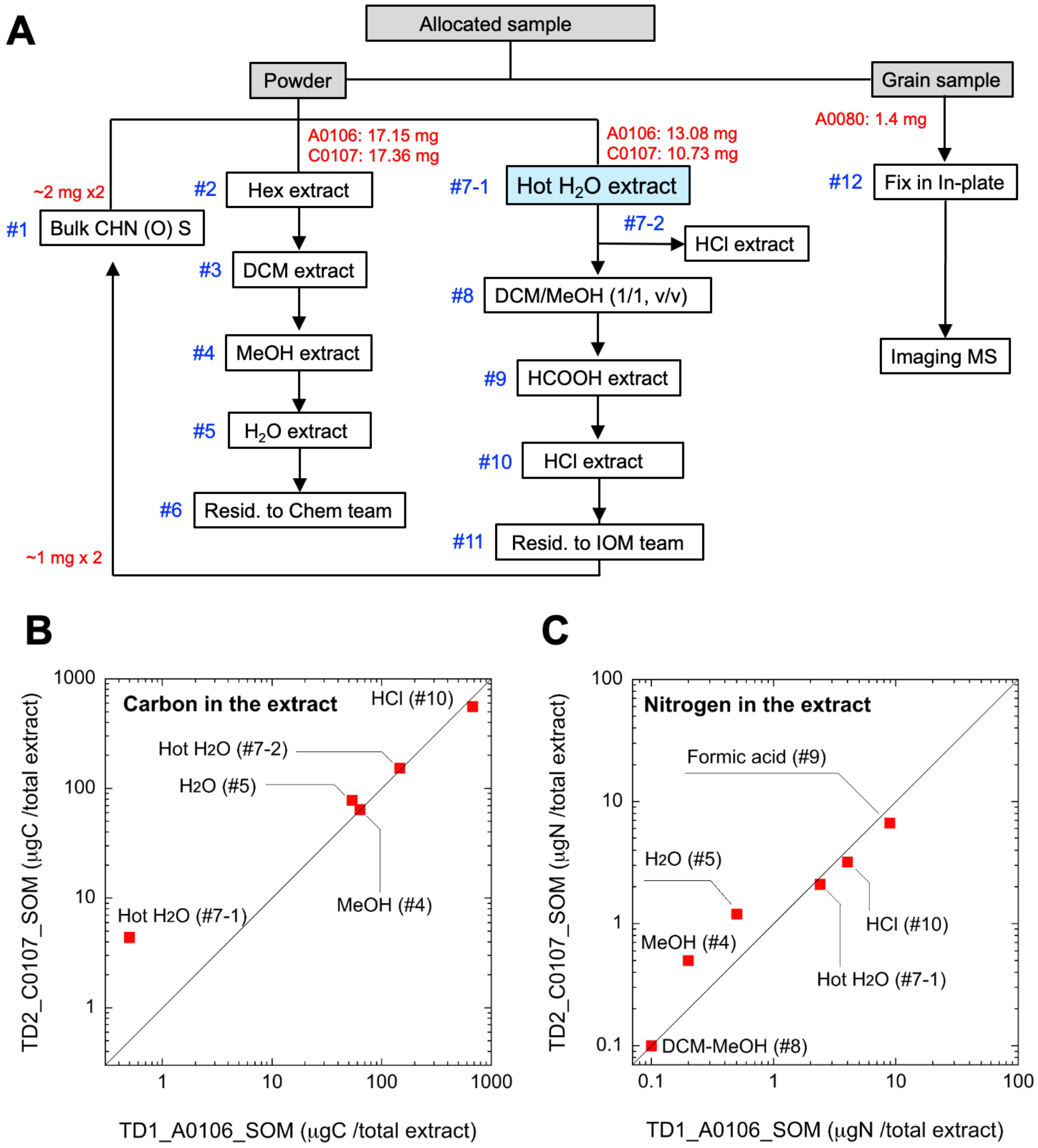

Supplementary Figure S3. Analytical scheme and sequential solvent extraction for Ryugu samples by the SOM team. (A) The extraction procedures were performed on an International Organization for Standardization (ISO)-5 (Class 100) clean bench inside an ISO-6 (Class 1000) clean room at Kyushu Univ. These extraction samples have been safely distributed to SOM team members and are being analyzed in detail at their laboratories [8]. The residues obtained from the insoluble solid fraction were also seamlessly transported to other initial analysis teams for further characterization [4,9]. **(B, C)** Carbon and nitrogen contents in the extracts from A0106 and C0107 for the process from #7 to #10 (Table S3). Carbon values are partially shown for data without solvent-derived blank effects. For example, carbon values of the formic acid fraction are not listed since completely drying the fraction is impossible.

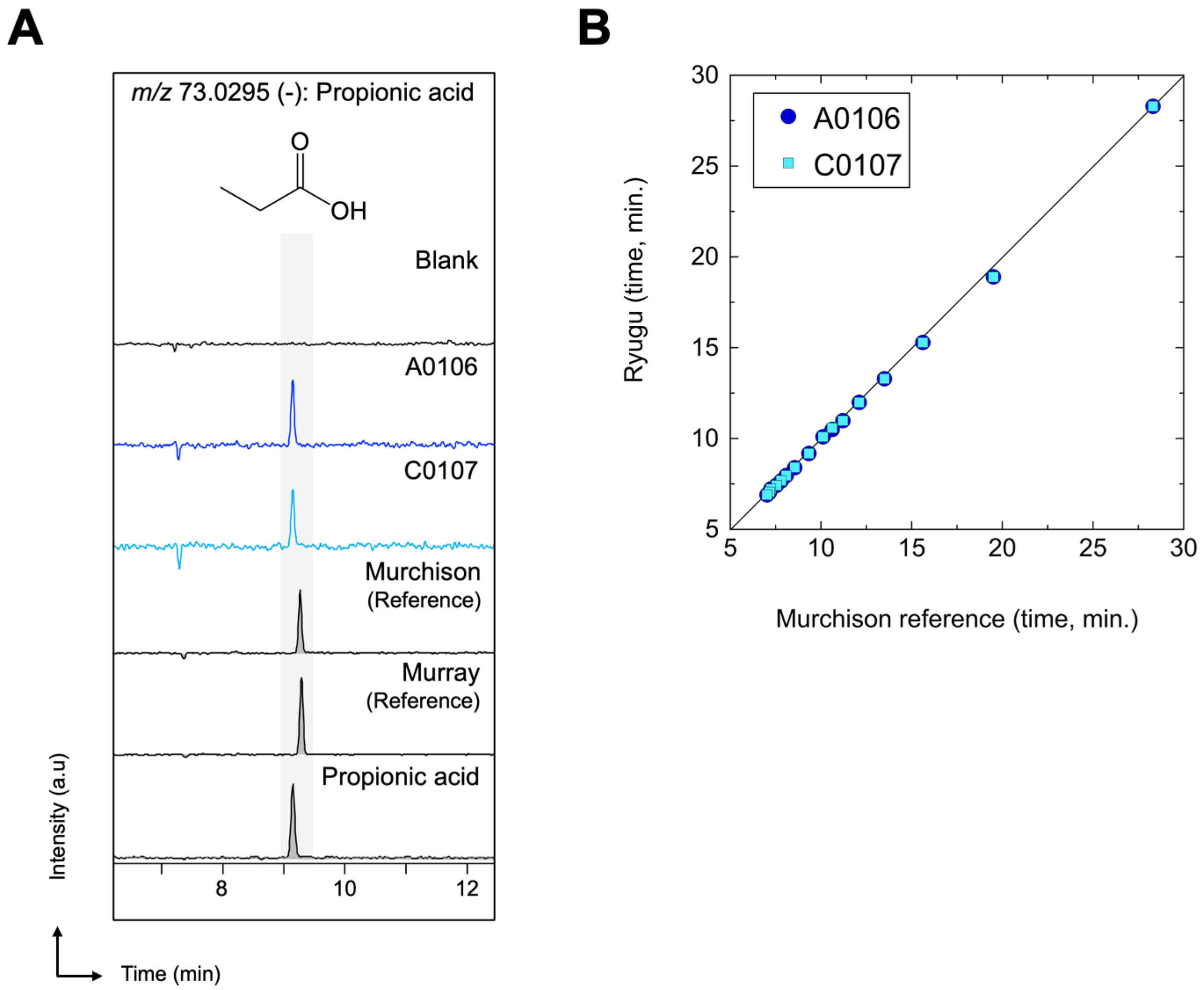

Supplementary Figure S4. Separation on CE-HRMS for Ryugu, carbonaceous reference standards (Murchison & Murray), and the carboxylic acid standard.

(A) High-resolution mass electropherograms of Ryugu (A0106 and C0107), Murchison, Murray and propionic acid standard. **(B)** Verification of migration time on high-resolution mass electropherograms for carboxylic acids (mono-, di-, and carboxylic acids of alkyl-straight chain molecules < C_{10}) between the Murchison reference and Ryugu sample (A0106 and C0107) on the theoretical 1:1 line. Ryugu A0106 and C0107 are indicated by blue and light blue, respectively. For more information, please see the references [17,35,36].

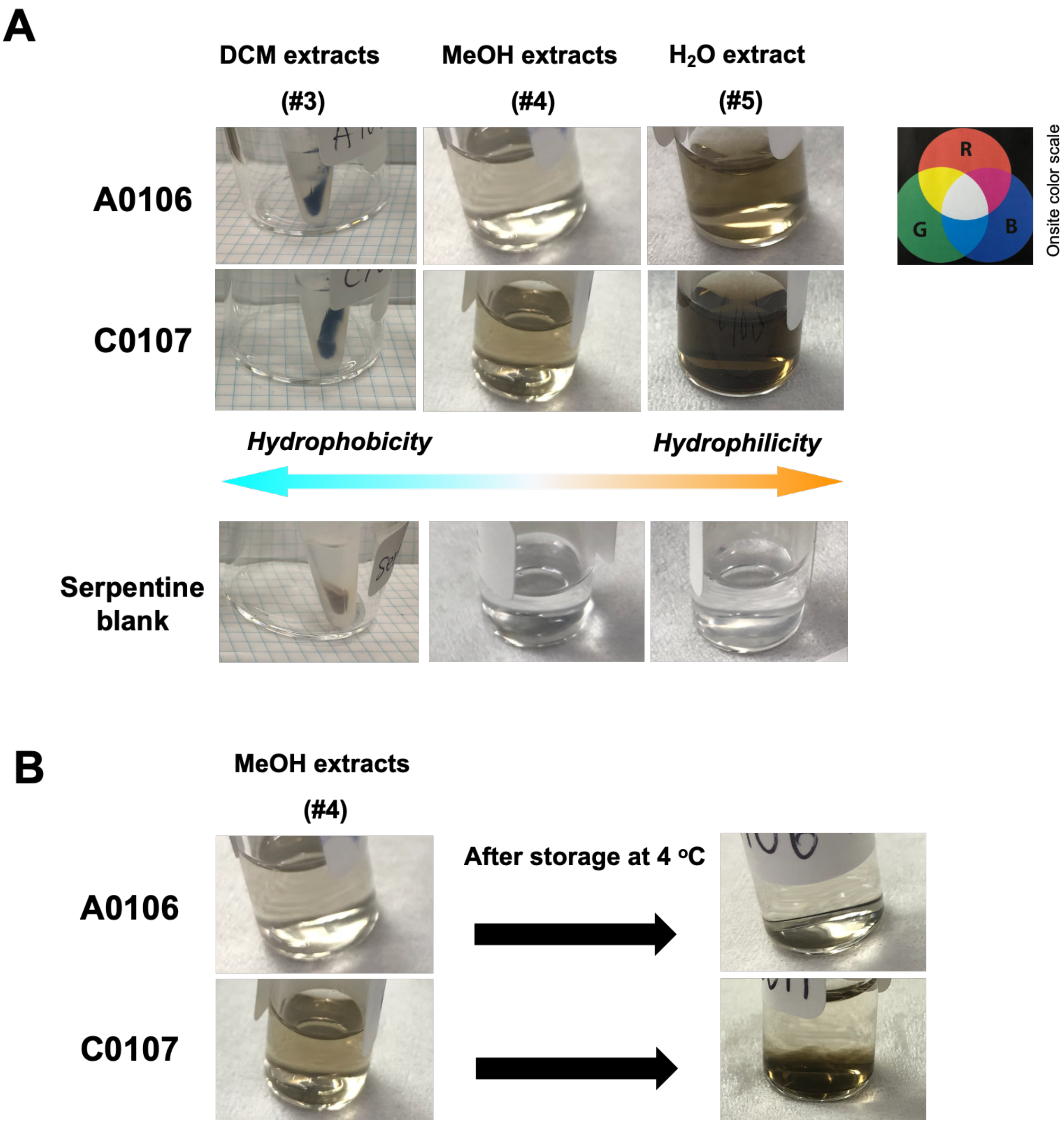

Supplementary Figure S5. Representative colors of extracts and colloids in the supernatant observed during sequential solvent extraction for the Ryugu aggregate samples (A0106 and C0107).

(A) Brownish colloidal colors (#4 MeOH extract, #5 water extract: each 600 µL scale) were observed for A0106 and C0107 [8,13,17]. The onsite color reference is shown in red (R), green (G), and blue (B) [16]. The soluble molecular survey data of formic acid extract #9 and the nitrogen isotopic profiles are shown in Figure S7 and Figure S8, respectively. **(B)** Colloidal precipitation was observed in the methanol extract (each 600 µL) and refrigerated storage at 4 °C. The photo images were taken during sequential extraction in a cleanroom at Kyushu Univ [8].

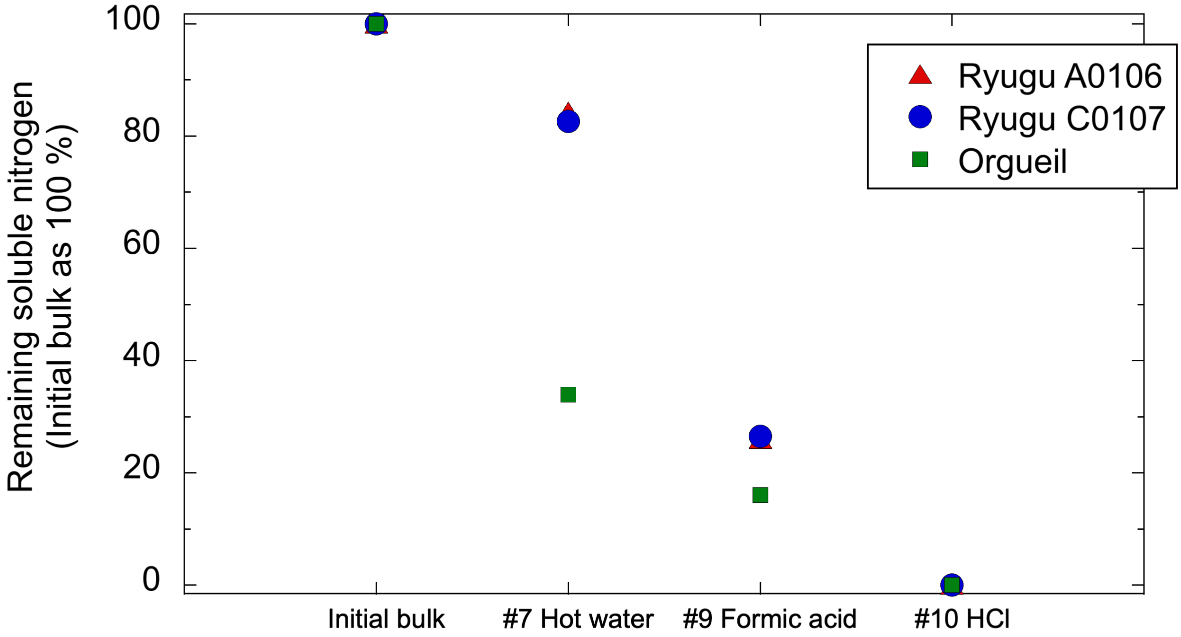

Supplementary Figure S6. Soluble nitrogen from the initial bulk to the sequential extraction.

The fraction of SOM (f_{SOM}) normalized by the nitrogen-based relative abundance (%, as initial bulk to the procedures of #7, #9, and #10) is shown for Ryugu A0106, C0107 and the reference of Orugeil. Notably, we used an extraction solvent containing carbon (formic acid), and the carbon percentage (%) was not discussed (Table S4).

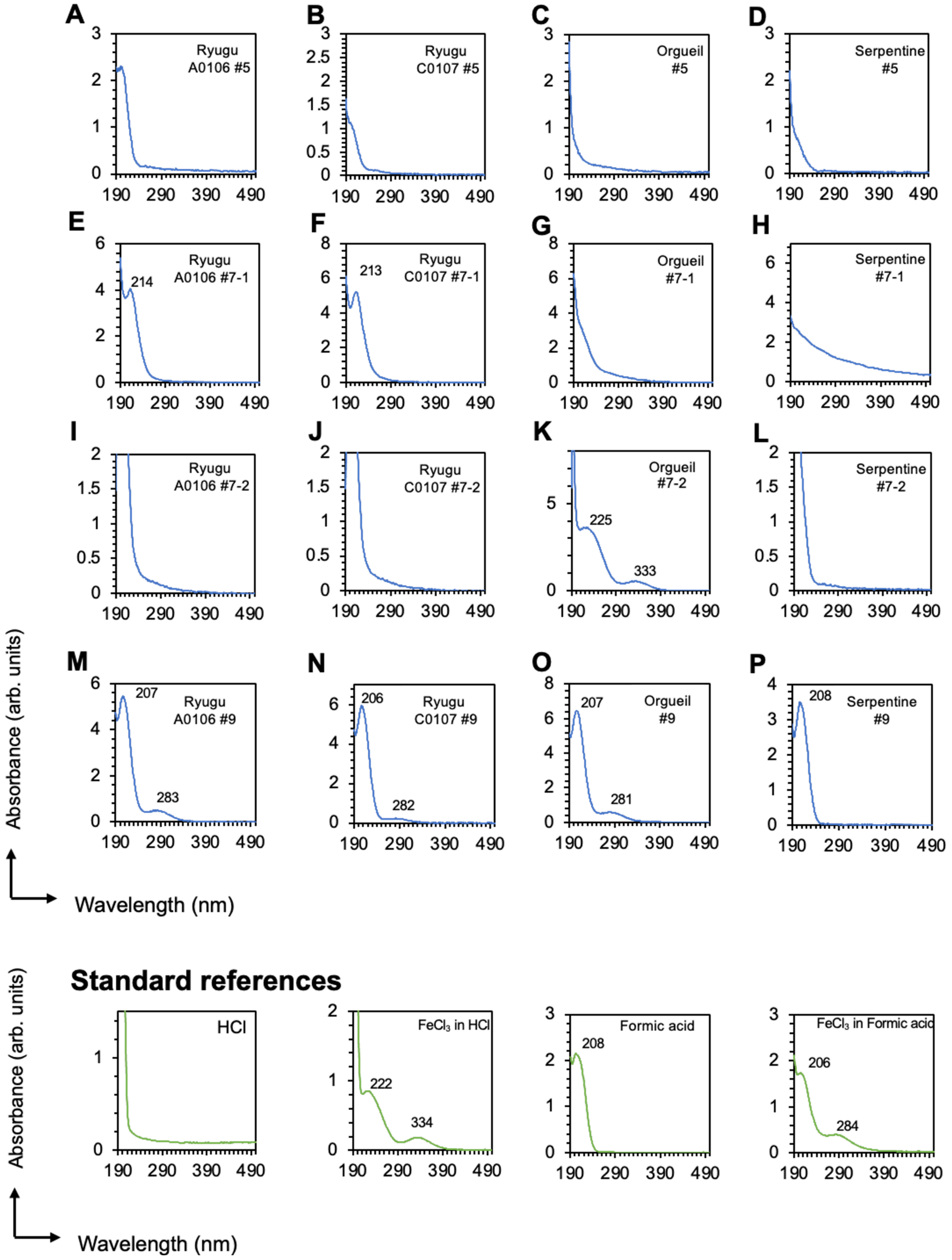

Supplementary Figure S7. UV–visible spectra of extracts from powders of the Ryugu samples (A0106 and C0107), the Orgueil meteorite, and thermally treated serpentine.

(**A–D**) H_2O extracts. (**E–H**) H_2O-HCl extracts. Please see the hot-water extracts fraction numbers of #7-1(unhydrolyzed) and #7-2 (hydrolyzed) [8, 13, 18, 19]. (**I–L**) HCl extracts. (**M–P**) Formic acid extracts with the reference aqueous solutions for HCl, 0.8 mM $FeCl_3$ in 1 M HCl, formic acid, and 0.16 mM $FeCl_3$ in 16 mM formic acid. The horizontal and vertical axes represent the wavelength (nm) and absorbance (arbitrary unit), respectively.

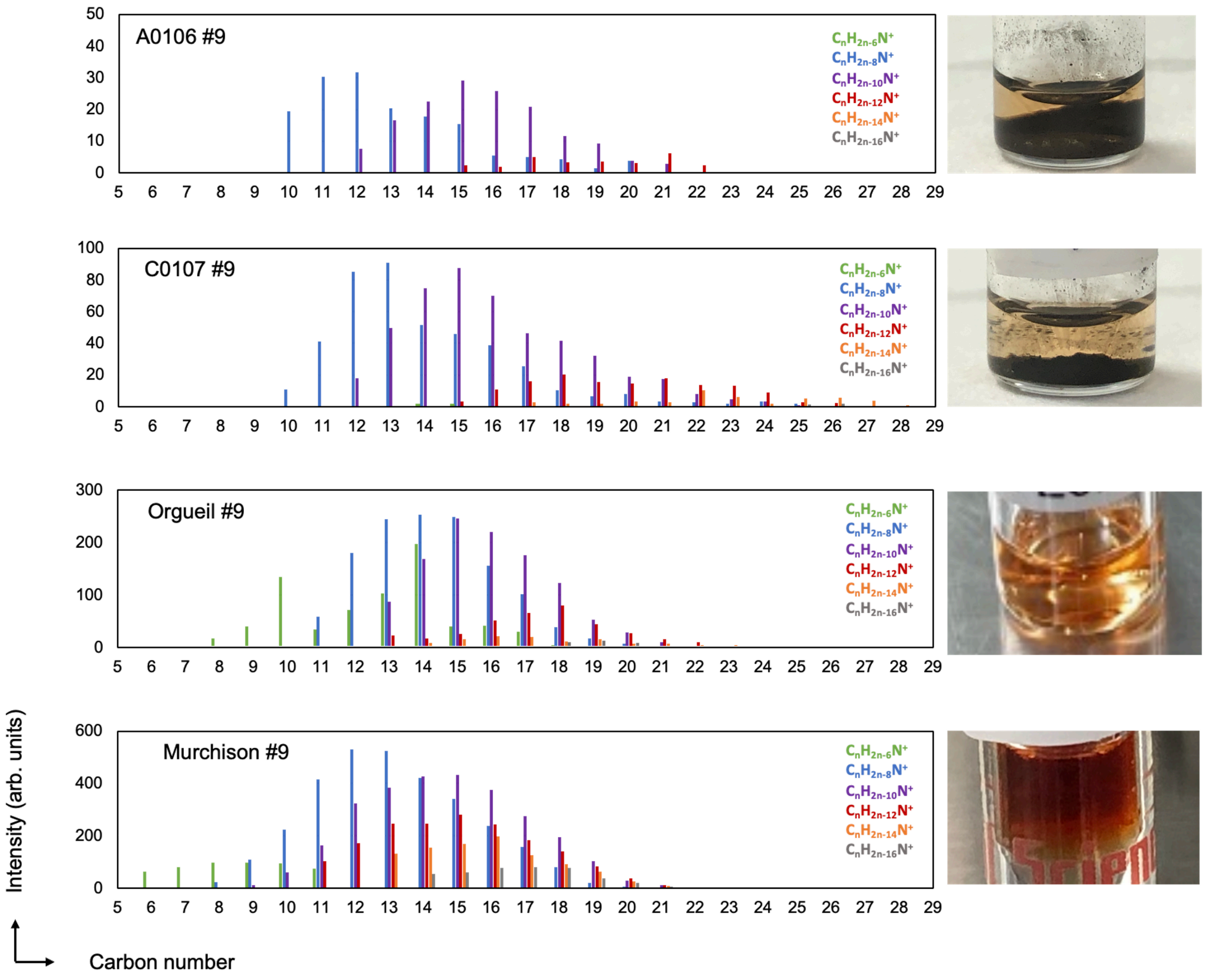

Supplementary Figure S8. Nitrogen-containing molecular compositions of formic acid extracts (#9). Based on a previous method [25], the nitrogen-containing molecular series are shown in diagrams (e.g., $C_nH_{2n-6}N^+$, $C_nH_{2n-8}N^+$, and $C_nH_{2n-10}N^+$) with comparisons among Ryugu (A0106 and C0107), Orgueil, and Murchison. Here, we note that the blank run of formic acid (purity >99%, FUJIFILM Wako Pure Chemical Corporation) was not significant during the sequential analysis (i.e., the instrumental background level of the SALDI system).

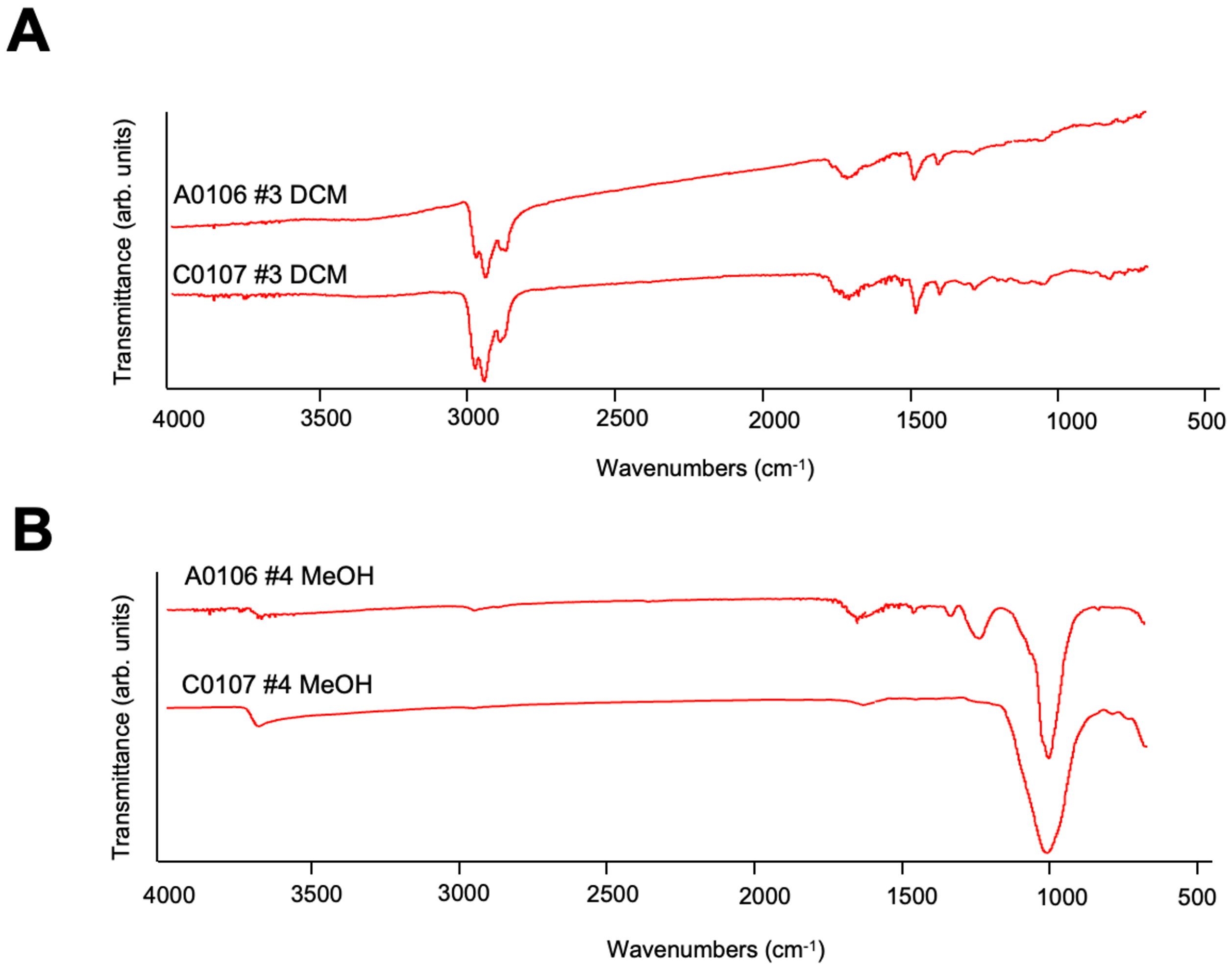

Supplementary Figure S9. Comparison of the FTIR spectra obtained for the solvent extracts from the Ryugu samples (A0106 and C0107).

(A) The DCM extract of C0107 presented an absorption band at 2850-2950 cm^{-1} (CH_2/CH_3 bonds), similar to that of A0106. **(B)** MeOH extracts. Vertical axis represents the transmittance in arbitrary units. Naraoka et al. (2023) reported preliminary data on A0106 profiles [8].

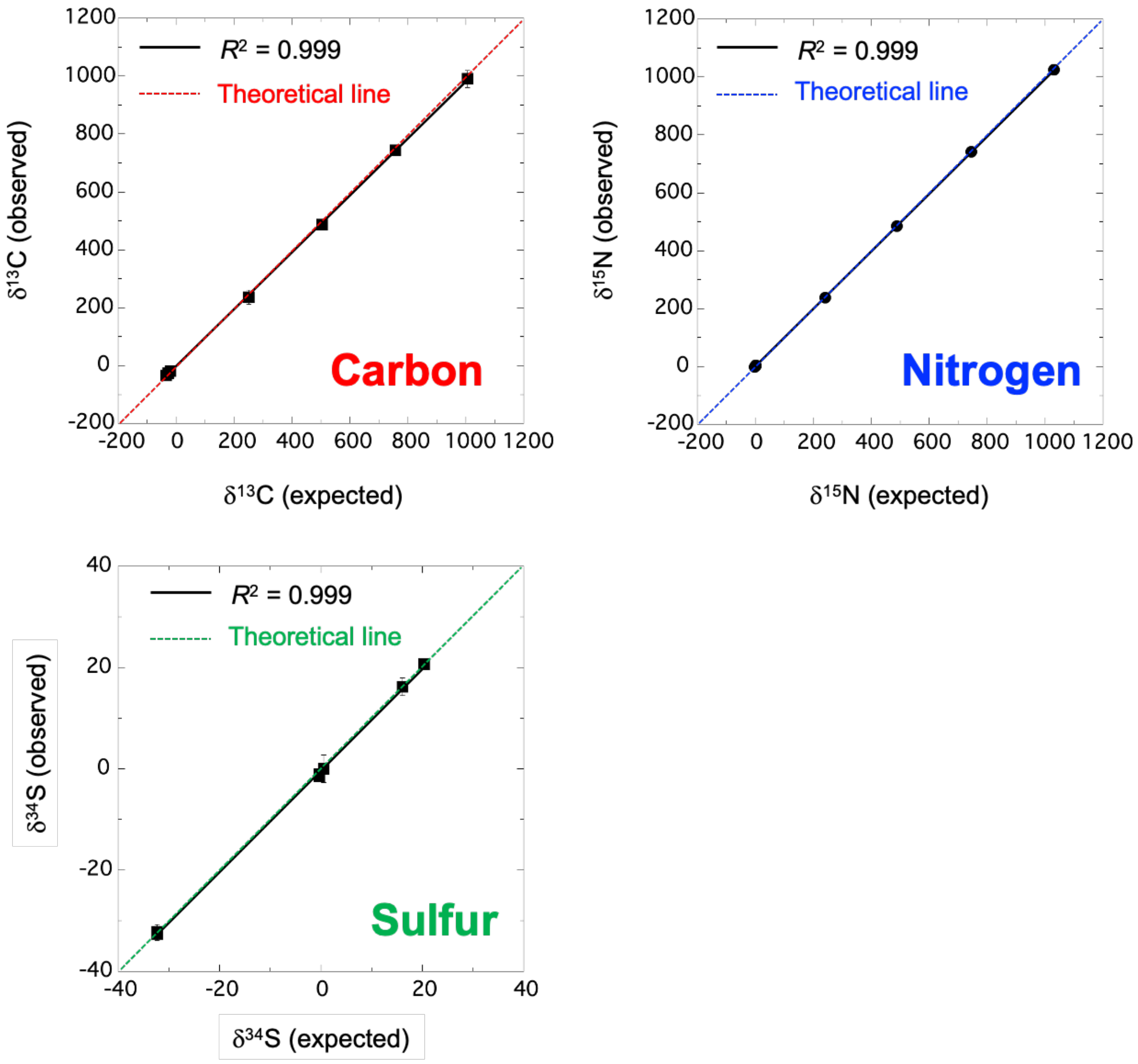

Supplementary Figure S10. Standardizing the analytical precision and accuracy of isotopic compositions. High-precision and high-accuracy analytical optimization using reference standards of carbon (C), nitrogen (N), and sulfur (S) covered a wide range of isotopic compositions. $\delta^{13}C$, ‰ vs. VPDB; within ^{13}C-enriched and ^{13}C-depleted profiles; $\delta^{15}N$, ‰ vs. Air; within ^{15}N-enriched and ^{15}N-depleted profiles. $\delta^{34}S$, ‰ vs. VCDT; within ^{34}S-enriched and ^{34}S-depleted profiles. The horizontal and vertical axes represent the nominal value (i.e., expected) and the measured value (i.e., observed), respectively [37,38].

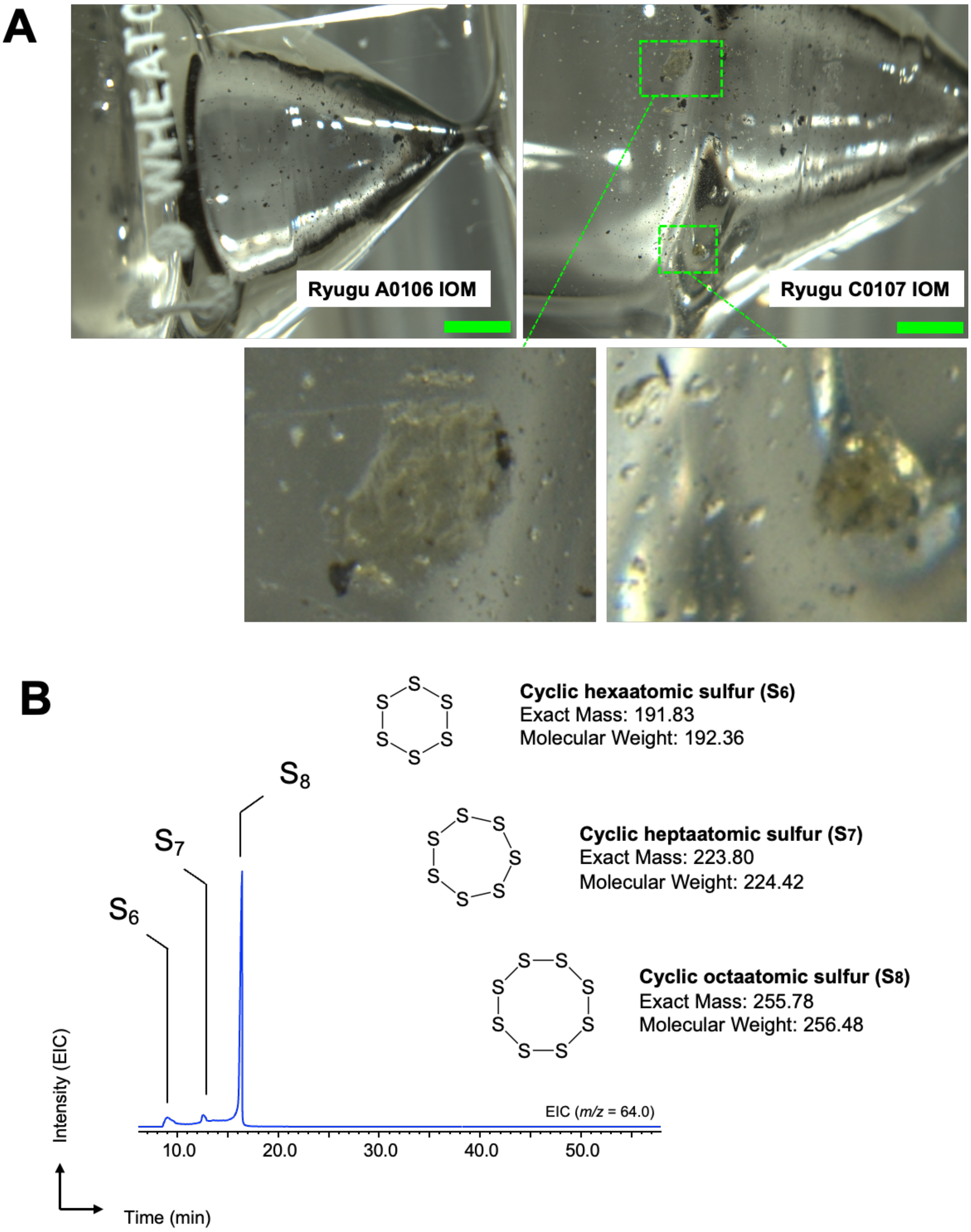

Supplementary Figure S11. Identification of cyclic sulfur molecules observed in the IOM leachate. (A) Photograph of IOM, suggesting the presence of a sticky yellowish precipitate. The scale bar stands for 200 μm. **(B)** Gas chromatographic separation of cyclic sulfur molecules (S_6, S_7, S_8). The corresponding mass fragmentations with the NIST references are shown in Figure S12. For the detection of S_8 in the soluble organic fraction, please see a previous report [20]. Notably, there are reports evaluating the presence and chemical form of sulfur-bearing species using scanning transmission electron microscopy-energy dispersive X-ray spectroscopy (STEM-EDS) [9] and XANES (X-ray absorption near edge structure) spectroscopy [39].

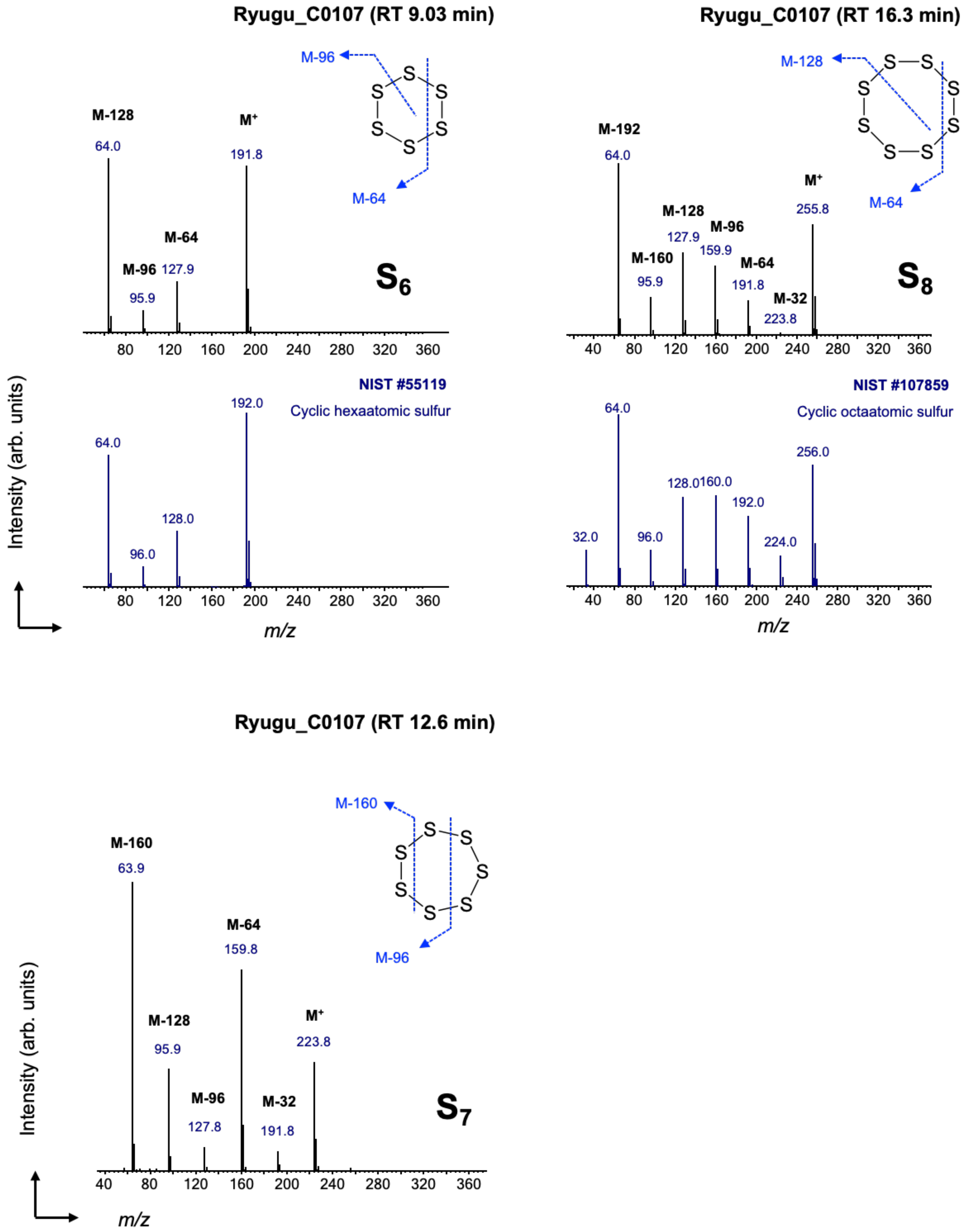

Supplementary Figure S12. Mass fragmentation of cyclic sulfur molecules observed in the IOM leachate. GC/MS analysis of the hexane extract from the IOM fraction to identify the cyclic sulfur molecules (S_6, S_7, S_8) and corresponding mass fragmentations with the NIST references (database ID #55119 for S_6, #107859 for S_8). For the detection of S_8 in the soluble organic fraction, please see the previous report [20].

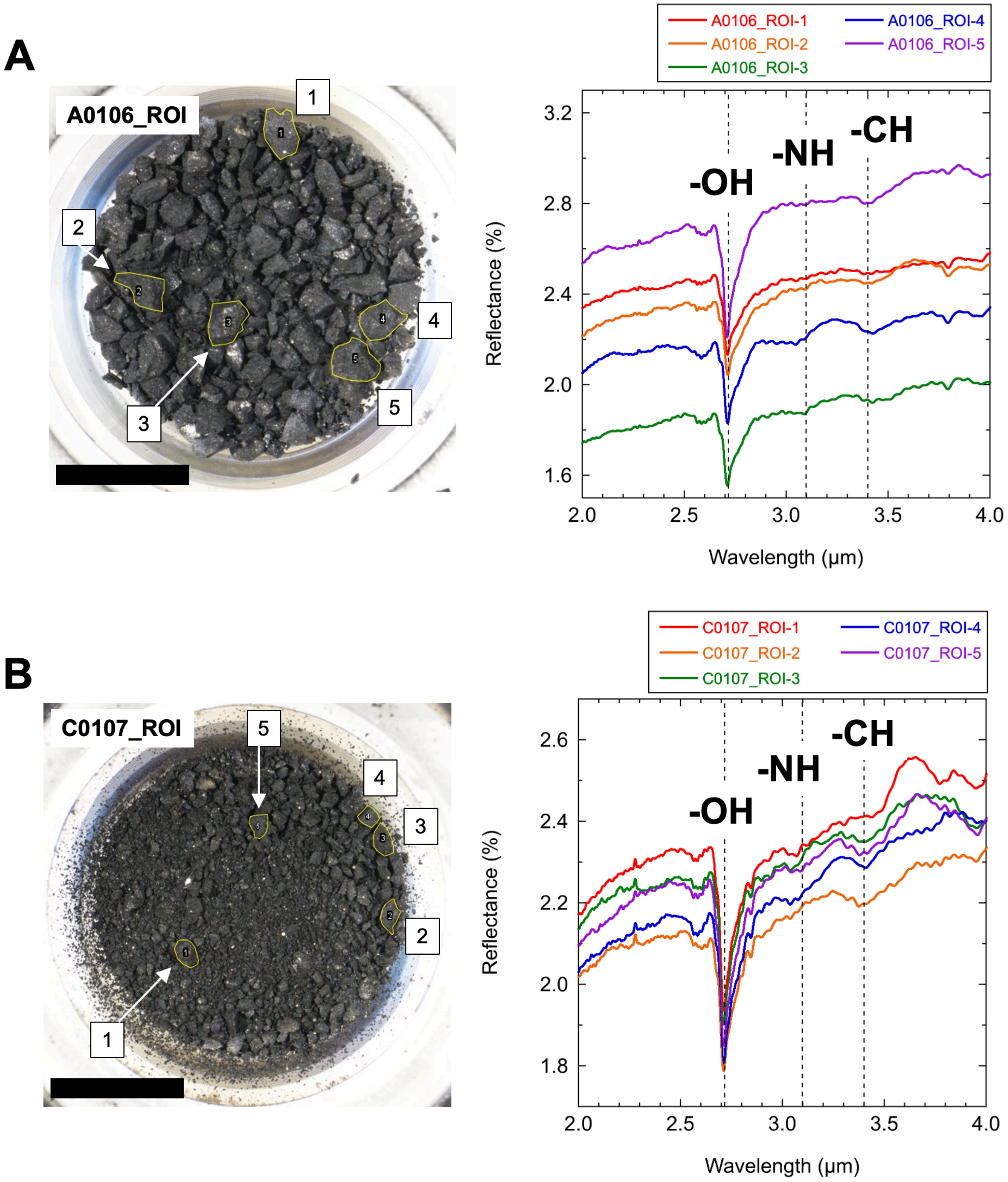

Supplementary Figure S13. Verification of the potential heterogeneity and homogeneity of the Ryugu samples (A0106 and C0107) in the grain-scale particles by FT-IR observation.

(A, B) Functional group-specific (-OH, -NH, -CH) signals were acquired according to the technical report [41], implying some partial heterogeneity in the ROIs. The scale bar stands for 3 mm. The partial organic heterogeneity was also shown on the mm scale [32].

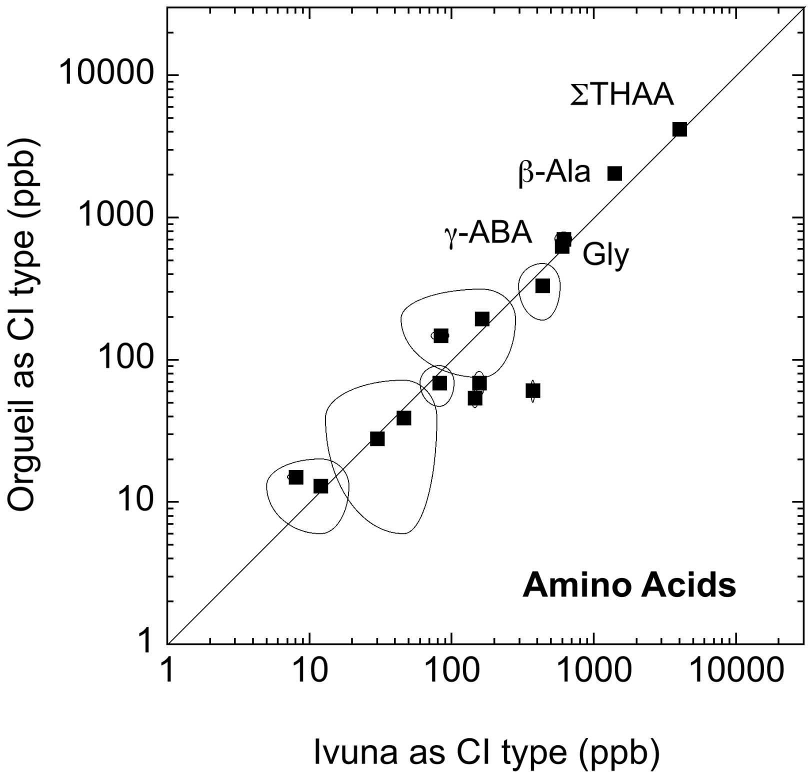

Supplementary Figure S14. Amino acids of the CI type for Ivuna and Orgueil.

Data compilation based on the reference for identified amino acids [41]. The data compilation is courtesy of the authors. The individual molecular information is shown in the diagram [41] with the following review [42].

Supplementary Table S1. Qualitative and quantitative results of hot water-extractable hydrophilic compounds (C, H, O, S) for hydroxy acids and other related molecules from Ryugu samples.

The reference data of CM meteorites (Murchison, Murray) are shown with the scale of parts per billion (ppb).

Name	Formula	Monoisotopic mass	Ryugu			CM group	
			A0106	C0107	Average	Murchison	Murray
**Hydroxy acid**							
Glycolic acid	$C_2H_4O_3$	76.01605	984.3	677.3	830.8	1011.8	874.6
Lactic acid	$C_3H_6O_3$	90.03169	2611.6	2775.8	2693.7	3223.7	3461.2
2-Hydroxybutyric acid	$C_4H_8O_3$	104.04734	321.7	309.8	315.7	640.4	839.0
2-Hydroxyisobutyric acid	$C_4H_8O_3$	104.04734	209.6	166.3	188.0	1051.7	1537.1
3-Hydroxybutyric acid	$C_4H_8O_3$	104.04734	1727.2	1772.2	1749.7	142.2	157.7
Glyceric acid	$C_3H_6O_4$	106.02661	202.1	167.4	184.8	151.4	203.4
2-Hydroxyvaleric acid	$C_5H_{10}O_3$	118.06300	437.0	370.2	403.6	1622.5	2461.3
3-Hydroxyisovaleric acid	$C_5H_{10}O_3$	118.06300	169.7	165.4	167.6	120.4	217.9
5-Hydroxypentanoic acid	$C_5H_{10}O_3$	118.06300	187.5	210.9	199.2	152.5	274.6
2-Hydroxy-4-methylvaleric acid	$C_6H_{12}O_3$	132.07864	446.6	426.7	436.7	1963.1	2568.9
o-Hydroxybenzoic acid	$C_7H_6O_3$	138.03169	213.4	578.1	395.8	467.8	746.4
Mevalonic acid	$C_6H_{12}O_4$	148.07356	72.9	92.0	82.4	543.5	628.3
**Di-carboxylic hydroxy acid**							
Malic acid	$C_4H_6O_5$	134.02153	167.3	64.0	115.6	484.6	778.4
2-Hydroxyglutaric acid	$C_5H_8O_5$	148.03717	156.4	109.1	132.8	545.6	782.4
Citramalic acid	$C_5H_8O_5$	148.03717	56.5	18.9	37.7	648.3	1208.1
2-Carboxybenzaldehyde	$C_8H_6O_3$	150.03169	19.5	29.0	24.2	33.0	27.5
10-Hydroxydecanoic acid	$C_{10}H_{20}O_3$	188.14125	110.5	173.6	142.0	345.3	589.5
**Di-carboxylic acid**							
Oxalic acid	$C_2H_2O_4$	89.99531	1278.0	907.1	1092.6	1881.3	906.0
Malonic acid	$C_3H_4O_4$	104.01096	60.6	52.6	56.6	289.7	244.9
Maleic acid	$C_4H_4O_4$	116.01096	201.3	273.6	237.5	686.2	306.9
Succinic acid	$C_4H_6O_4$	118.02661	1094.6	743.6	919.1	2587.4	2985.8
Citraconic acid	$C_5H_6O_4$	130.02661	1115.5	991.8	1053.7	1640.5	1477.0
Glutaric acid	$C_5H_8O_4$	132.04225	456.8	402.9	429.9	2032.1	2729.2
2-Oxoglutaric acid	$C_5H_6O_5$	146.02153	118.9	54.8	86.8	21.6	58.3
Adipic acid	$C_6H_{10}O_4$	146.05791	422.3	333.3	377.8	1535.0	2097.8
2-Oxoadipic acid	$C_6H_8O_5$	160.03717	23.4	21.2	22.3	34.0	70.0
Pimelic acid	$C_7H_{12}O_4$	160.03717	245.9	49.2	147.6	1040.9	1317.2
Suberic acid	$C_8H_{14}O_4$	174.08920	117.1	41.9	79.5	849.2	923.6
2-Isopropylmalic acid	$C_7H_{12}O_5$	176.06847	22.0	n.d.	22.0	773.6	1067.3
Azelaic acid	$C_9H_{16}O_4$	188.10486	67.2	130.1	98.6	645.0	687.7
Sebacic acid	$C_{10}H_{18}O_4$	202.12051	28.9	21.1	25.0	496.5	418.4
**Tricarboxylic acid**							
Citric acid	$C_6H_8O_7$	192.02701	188.1	101.9	145.0	25.4	32.0
**Other carboxylic acid and isomers**							
Propionic acid	$C_3H_6O_2$	74.03678	684.0	462.2	573.1	646.69	1210.7
Butyric acid<br>Isobutyric acid	$C_4H_8O_2$	88.05243	986.0	671.4	828.7	1696.05	2252.0
Hexanoic acid	$C_6H_{12}O_2$	116.08373	1277.8	1103.7	1190.7	3427.88	10544.2
Heptanoic acid	$C_7H_{14}O_2$	130.09938	636.4	536.1	586.2	1879.02	4645.7
Octanoic acid	$C_8H_{16}O_2$	144.11504	1607.0	2512.4	2059.7	4404.31	10115.6
Pelargonic acid	$C_9H_{18}O_2$	158.13068	1471.2	1806.0	1638.6	5832.66	9921.9
Tiglic acid	$C_5H_8O_2$	100.05243	38.3	55.2	46.7	50.2	61.2
Pyruvic acid	$C_3H_4O_3$	88.01605	304.4	57.6	181.0	235.1	1415.2
4-Oxovaleric acid	$C_5H_8O_3$	116.04734	497.2	404.8	451.0	768.8	933.2
5-Oxohexanoic acid	$C_6H_{10}O_3$	130.06299	247.2	174.1	210.7	994.4	1005.5
Isethionic acid	$C_2H_6O_4S$	125.99868	365.7	603.5	484.6	531.3	266.3
3-Phenylpropionic acid	$C_9H_{10}O_2$	150.06809	67.3	86.2	76.8	254.2	529.4
Cumic acid	$C_{10}H_{12}O_2$	164.08373	17.8	26.9	22.3	323.5	587.7
Phthalic acid	$C_8H_6O_4$	166.02661	1376.4	1100.5	1238.4	2725.5	2317.2
Tropic acid	$C_9H_{10}O_3$	166.06299	104.7	130.6	117.7	1582.3	315.8
Ferulic acid	$C_{10}H_{10}O_4$	194.05791	37.5	65.3	51.4	1033.5	87.6
Isovaleric acid<br>+ 2-Methylbutyric Acid<br>+ Valeric acid	$C_5H_{10}O_2$	102.06808	994.7	759.3	877.0	2456.74	3996.9
p-Toluic acid<br>+ o-Toluic acid<br>+ m-Toluic acid	$C_8H_8O_2$	136.05243	62.5	136.2	99.3	191.1	328.5
m-Hydroxybenzoic acid<br>+ p-Hydroxybenzoic acid	$C_7H_6O_3$	138.03169	30.6	95.8	63.2	29.9	43.7
Hydroxyphenylacetic acid<br>+ Mandelic acid	$C_8H_8O_3$	152.04735	42.2	53.3	47.8	70.6	130.2
8-Hydroxyoctanoic acid<br>2-Hydroxyoctanoic acid	$C_8H_{16}O_3$	160.10994	281.1	284.7	282.9	958.7	2460.6
8-Hydroxyoctanoic acid<br>2-Hydroxyoctanoic acid	$C_8H_{16}O_3$	160.10994	152.6	174.1	163.4	382.6	1130.5
o-Coumaric acid<br>+ *p*-Coumaric acid	$C_9H_8O_3$	164.04735	35.9	33.9	34.9	94.3	142.8

Supplementary Table S2. Qualitative and quantitative results of hot water-extractable hydrophilic compounds (C, H, N) for hydroxy acids and other related molecules from Ryugu samples.

The reference data of CM meteorites (Murchison, Murray) are shown with the scale of parts per billion (ppb). Notably, the quantitative errors of the organic acid mixed solution (average mass number: 120.2 ± 40.6, *n* = 5) and amino acid mixed solution (average mass number: 132.2 ± 31.0, *n* = 27) were better than 94.9 ± 9.4% (*n* = 5) and 90.4 ± 12.3% (*n* = 27), respectively, on the accuracy of the analytical conditions.

Name	Formula	Monoisotopic mass	Ryugu			CM group	
			A0106	C0107	Average	Murchison	Murray
**N-containing molecules**							
Urea	CH_4N_2O	60.03236	1511.1	1942.6	1726.8	618.7	399.6
C1-Urea (methy-urea)	$C_2H_6N_2O$	74.04801	88.9	168.8	128.8	138.1	104.2
C2-Urea	$C_3H_8N_2O$	88.06366	228.4	269.3	248.8	37.4	0.0
C3-Urea	$C_4H_{10}N_2O$	102.07932	991.9	717.7	854.8	67.3	31.7
C4-Urea	$C_5H_{12}N_2O$	116.09496	1636.5	1421.2	1528.8	98.6	108.5
C5-Urea	$C_6H_{14}N_2O$	130.11061	244.2	185.0	214.6	31.1	44.1
C6-Urea	$C_7H_{16}N_2O$	144.12627	381.3	442.5	411.9	82.0	140.8
C7-Urea	$C_8H_{18}N_2O$	158.14191	177.3	n.a.	177.3	75.6	141.5
Benzimidazole	$C_7H_6N_2$	118.05310	9.7	7.5	8.6	41.8	61.0
Quinoline	C_9H_7N	129.05785	33.6	21.2	27.4	25.7	36.7
Isoquinoline	C_9H_7N	129.05785	25.7	23.8	24.7	9.8	16.5
Hydroxyindole	C_8H_7NO	133.05277	n.d.	29.9	29.9	10.2	19.1
5,6-Dihydroxyindole	$C_8H_7NO_2$	149.04768	20.9	26.3	23.6	20.7	12.6
N-Acetyl-β-alanine	$C_5H_9NO_3$	131.05824	28.5	33.8	31.1	82.7	82.4
N-Acetylglycine	$C_4H_7NO_3$	117.04260	61.9	43.5	52.7	32.2	16.9
Guanidoacetic acid	$C_3H_7N_3O_2$	117.05383	2.9	9.1	6.0	14.7	5.5
6-Hydroxynicotinic acid	$C_6H_5NO_3$	139.02695	55.9	90.4	73.1	35.81	63.0
4-Acetamidobutanoic acid	$C_6H_{11}NO_3$	145.07390	47.5	19.2	33.3	163.1	141.0
Isovalerylalanine	$C_8H_{15}NO_3$	173.10519	46.1	n.d.	46.1	212.5	194.6

n.d.: not detected
n.a.: not available (detected but not quantified)

Supplementary Table S3. Summary of the data obtained for carbon, nitrogen, hydrogen, oxygen, and sulfur contents (wt %) with their stable isotopic compositions for the Ryugu IOM solid residues.
For the initial bulk data (IB) of Ryugu for A0106 and C0107, please see the references [8,13,17].

Ryugu A0106	Shape	Weight (μg)	Carbon (wt%)	1σ	$\delta^{13}C$ (‰ vs. VPDB)	1σ	Nitrogen (wt%)	1σ	$\delta^{15}N$ (‰ vs. Air)	1σ	weight C/N ratio	1σ
A0106_IOM_#1	small grains	9.8	67.1		-16.7		2.80		27.4		24.1	
A0106_IOM_#2	small grains	8.0	35.2		-17.2		1.50		30.7		23.0	
**Average (*n* = 2)**		**8.9**	**51.2**	**±22.6**	**-17.0**	**±0.4**	**2.15**	**±0.92**	**29.1**	**±2.3**	**23.6**	**±0.8**
			**Hydrogen (wt%)**	**1σ**	**δD (‰ vs. VSMOW)**	**1σ**	**Oxygen (wt%)***	**1σ**	**$\delta^{18}O$ (‰ vs. VSMOW)**	**1σ**	**weight O/H ratio**	**1σ**
A0106_IOM_#3	small grains	22.8	6.6		90		13.1		21.9		2.0	
A0106_IOM_#4	small grains	15.5	4.9		283		10.8		15.6		2.2	
A0106_IOM_#5	small grains	7.3	5.1		142		10.9		22.5		2.1	
**Average (*n* = 3)**		**15.2**	**5.5**	**±0.93**	**172**	**±99.9**	**11.6**	**±1.30**	**20.0**	**±3.8**	**2.1**	**±0.1**
			**Sulfur (wt%)**	**1σ**	**$\delta^{34}S$ (‰ vs. VCDT)**	**1σ**					**weight C/S ratio**	**1σ**
A0106_IOM_#6	small grains	9.7	18.0		0.4						-	
A0106_IOM_#7	small grains	8.4	14.8		2.1						-	
**Average (*n* = 3)**		**9.1**	**16.4**	**±2.26**	**1.2**	**±1.24**				**Average**	**3.12**	

Ryugu C0107	Shape	Weight (μg)	Carbon (wt%)	1σ	$\delta^{13}C$ (‰ vs. VPDB)	1σ	Nitrogen (wt%)	1σ	$\delta^{15}N$ (‰ vs. Air)	1σ	weight C/N ratio	1σ
C0107_IOM_#1	small grains	5.7	30.9		-18.7		1.4		25.5		21.6	
C0107_IOM_#2	small grains	7.0	28.1		-17.8		1.2		30.9		23.6	
**Average (*n* = 2)**		**6.4**	**29.5**	**±2.0**	**-18.3**	**±0.6**	**1.30**	**±0.14**	**28.2**	**±3.8**	**22.6**	**±1.4**
			**Hydrogen (wt%)**	**1σ**	**δD (‰ vs. VSMOW)**	**1σ**	**Oxygen (wt%)***	**1σ**	**$\delta^{18}O$ (‰ vs. VSMOW)**	**1σ**	**weight O/H ratio**	**1σ**
C0107_IOM_#3	small grains	18.8	5.0		237.0		9.6		22.4		1.9	
C0107_IOM_#4	small grains	52.0	4.9		280.0		9.9		25.2		2.0	
**Average (*n* = 2)**		**35.4**	**5.0**	**±0.07**	**259**	**±30.4**	**9.8**	**±0.21**	**23.8**	**±2.0**	**2.0**	**±0.1**
			**Sulfur (wt%)**	**1σ**	**$\delta^{34}S$ (‰ vs. VCDT)**	**1σ**					**weight C/S ratio**	**1σ**
C0107_IOM_#5	small grains	7.1	64.3		-1.0						-	
C0107_IOM_#6	small grains	4.4	50.4		0.2						-	
C0107_IOM_#7	small grains	2.6	53.6		0.9						-	
**Average (*n* = 3)**		**4.7**	**56.1**	**±7.25**	**0.0**	**±0.95**				**Average**	**0.53**	

*Pyrolyzed oxygen released at 1400 °C under a helium gas flow as described [8].

Supplementary Table S4. Summary of the data obtained for carbon and nitrogen content and the isotopic compositions by solvent extraction for Ryugu (A0106 and C0107) and the reference (CI-Orgueil). The total extract volume was 600 µL for each solvent extraction [8]. Since we used an extraction solvent containing carbon (e.g., formic acid), the remaining carbon information is not shown below.

Name	Solvent	Sample code	Inj vol. µL	$\delta^{15}N$ ‰ (‰ vs. Air)	$\delta^{13}C$ ‰ (‰ vs. VPDB)	Total N µg/extr	Total C µg/extr	wt C/N	atm C/N
**Ryugu**									
**A106**	Methanol exract	SOM-#4 MeOH	10	-	-21.9	0.2	63.5	266.0	310.3
**C107**			10	-	-20.6	0.5	64.2	122.2	142.5
**A106**	H_2O exract	SOM-#5 H_2O fraction	40	27.9	-24.3	0.5	53.7	113.2	132.0
**C107**			40	24.2	-23.0	1.2	78.1	63.6	74.2
**A106**	Hot H_2O exract	SOM-#7-1 Hot H_2O	10	63.1	-	2.4	0.5	0.2	0.3
**C107**			10	55.2	-	2.1	4.4	2.1	2.5
**A106**	DCM-MeOH exract	SOM-#8 DCM-MeOH	10	-	-	< 0.1	-	-	-
**C107**			9	-	-	< 0.1	-	-	-
**A106**	Formic acid exract	SOM-#9 Formic acid	5	3.6	-	8.9	-	-	-
**C107**			5	8.4	-	6.7	-	-	-
**A106**	HCl exract	SOM-#10 HCl	10	3.2	-26.3	4.0	676.8	168.2	196.2
**C107**			10	-2.5	-26.4	3.2	559.5	173.4	202.3
**Orgueil**									
	Methanol	SOM-#4 MeOH	10	47.6	-17.9	1.5	34.5	22.5	26.3
	Hot H2O	SOM-#7-1 Hot H_2O	10	25.2	-	7.8	17.0	2.2	2.5
	DCM-MeOH	SOM-#8 DCM-MeOH	10	-	-		3.4	-	-
	Formic acid	SOM-#9 Formic acid	10	6.8	-	2.1	-	-	-
	HCl	SOM-#10 HCl	10	2.5	-	1.9	-	-	-

Supplementary References

1. Tachibana, S. et al. Pebbles and sand on asteroid (162173) Ryugu: On-site observation and returned particles from two landing sites. *Science* **375**, 1011–1016 (2022).
2. Yada, T. et al. Preliminary analysis of the Hayabusa2 samples returned from C-type asteroid Ryugu. *Nature Astron.* **6**, 214–220 (2022).
3. Pilorget, C. et al. First compositional analysis of Ryugu samples by the MicrOmega hyperspectral microscope. *Nature Astron.* **6**, 221–225 (2022).
4. Yokoyama, T. et al. Samples returned from the asteroid Ryugu are similar to Ivuna-type carbonaceous meteorites. *Science* **379**, eabn7850 (2023).
5. Nakamura, T. et al. Formation and evolution of carbonaceous asteroid Ryugu: Direct evidence from returned samples. *Science* **379**, eabn8671 (2023).
6. Noguchi, T. et al. A dehydrated space-weathered skin cloaking the hydrated interior of Ryugu. *Nature Astron.* **7**, 170–181 (2023).
7. Okazaki, R. et al. Noble gases and nitrogen in samples of asteroid Ryugu record its volatile sources and recent surface evolution. *Science* **379**, eabo0431 (2023).
8. Naraoka, H. et al. Soluble organic molecules in samples of the carbonaceous asteroid (162173) Ryugu. *Science* **379**, abn9033 (2023).
9. Yabuta, H. et al. Macromolecular organic matter in samples of the asteroid (162173) Ryugu. *Science* **379**, eabn9057 (2023).
10. Ito, M. et al. A pristine record of outer Solar System materials from asteroid Ryugu's returned sample. *Nature Astron.* **6**, 1163–1171 (2022).
11. Nakamura, E. et al. On the origin and evolution of the asteroid Ryugu: A comprehensive geochemical perspective. *Proc. Jpn. Acad. Ser. B* **98**, 227–282 (2022).
12. Potiszil, C. et al. Insights into the formation and evolution of extraterrestrial amino acids from the asteroid Ryugu. *Nature Commun.* **14**, Article number: 1482 (2023).
13. Yoshimura, T. et al. Chemical evolution of primordial salts and organic sulfur molecules in the asteroid (162173) Ryugu. *Nature Commun.* **14**, Article number: 5284 (2023).
14. Naraoka, H. & Hashiguchi, M. Distinct distribution of soluble N-heterocyclic compounds between CM and CR chondrites. *Geochem. J.* **53**, 33–40 (2019).
15. Oba, Y. et al. Identifying the wide diversity of extraterrestrial purine and pyrimidine nucleobases in carbonaceous meteorites. *Nature Commun.* **13**, Article number: 2008 (2022).
16. Takano, Y. et al. CNHOS contents with their isotopic compositions and preliminary organic profiles from the Hayabusa2 samples. *Hayabusa symposium*, # Abstract S3-7 (2021).
17. Oba, Y. et al. Uracil in the carbonaceous asteroid (162173) Ryugu. *Nature Commun.* **14**, Article number: 1292 (2023a).
18. Parker, E.T. et al. Extraterrestrial amino acids and amines identified in asteroid Ryugu samples returned by the Hayabusa2 mission. *Geochim. Cosmochim. Acta* **347**, 42–57 (2023).

19. Furusho, A. et al. Enantioselective Three-Dimensional High-Performance Liquid Chromatographic Determination of Amino Acids in the Hayabusa2 Returned Samples from the Asteroid Ryugu. *J. Chromatogr. Open.*, doi:10.1016/j.jcoa.2024.100134 (2024).
20. Aponte, J.C. et al. PAHs, hydrocarbons, and dimethylsulfides in Asteroid Ryugu samples A0106 and C0107 and the Orgueil (CI1) meteorite. *Earth Planet Space* **75**, Article number: 28 (2023).
21. Zeichner, S. S. et al. Polycyclic aromatic hydrocarbons in samples of Ryugu formed in the interstellar medium. *Science* **382**, 1411–1416 (2023).
22. Ehrenfreund, P. & Charnley, S.B. Organic molecules in the interstellar medium, comets, and meteorites: A voyage from dark clouds to the early Earth. *Ann. Rev. Astron. Astrophys.* **38**, 427–483 (2000).
23. Sephton, M.A. Organic compounds in carbonaceous meteorites. *Natural Product Reports* **19**, 292–311 (2002).
24. Peral, F. & Gallego, E. Self-association of imidazole and its methyl derivatives in aqueous solution. A study by ultraviolet spectroscopy. *J. Mole. Structure* **415**, 187–196 (1997).
25. Furukawa, Y. et al. Distributions of CHN compounds in meteorites record organic syntheses in the early solar system. *Sci. Rep.* **13**, Article number: 6683 (2023).
26. Naraoka, H., Yamashita, Y., Yamaguchi, M. & Orthous-Daunay, F.-R. Molecular evolution of N-containing cyclic compounds in the parent body of the Murchison meteorite. *ACS Earth Space Chem.* **1**, 540–550 (2017).
27. Draine, B. & Lee, H.M. Optical properties of interstellar graphite and silicate grains. *Astrophys. J.* **285**, 89–108 (1984).
28. Peeters, E. et al. The rich 6 to 9 um spectrum of interstellar PAHs. *Astron. & Astrophys.* **390**, 1089–1113 (2002).
29. Turner, A.M. & Kaiser, R.I. Exploiting Photoionization Reflectron Time-of-Flight Mass Spectrometry to Explore Molecular Mass Growth Processes to Complex Organic Molecules in Interstellar and Solar System Ice Analogs. *Accounts Chem. Res.* **53**, 2791–2805 (2020).
30. Zhang, M. et al. Temperature dependence of IR absorption of hydrous/hydroxyl species in minerals and synthetic materials. *Ame. Mineral.* **92**, 1502–1517 (2007).
31. Oba, Y., Takano, Y., Dworkin, J.P. & Naraoka, H. Ryugu asteroid sample return provides a natural laboratory for primordial chemical evolution. *Nature Commun.* **14**, Article number: 3107 (2023b).
32. Hashiguchi, M. et al. The spatial distribution of soluble organic matter and their relationship to minerals in the asteroid (162173) Ryugu. *Earth Planet Space* **75**, Article number: 73 (2023).
33. Orthous- Daunay, F. R. et al. Molecular growth pattern of soluble CHN compounds from Ryugu. *Lunar and Planetary Science Conference (LPSC)*, #2367 (2022).
34. Schmitt-Kopplin, P. et al. Soluble organic matter Molecular atlas of Ryugu reveals cold hydrothermalism on C-type asteroid parent body. *Nature Commun.* **14**, Article number: 6525 (2023).
35. Sasaki, K. et al. Metabolomics platform with capillary electrophoresis coupled with high-resolution mass spectrometry for plasma analysis. *Anal. Chem.* **91**, 1295–1301 (2019).
36. Sugimoto, M., Wong, D. T., Hirayama, A., Soga, T. & Tomita, M. Capillary electrophoresis mass

spectrometry-based saliva metabolomics identified oral, breast and pancreatic cancer–specific profiles. *Metabolomics* **6**, 78–95 (2010).

37. Ogawa, N.O. et al. Ultra-sensitive elemental analyzer/isotope ratio mass spectrometer for stable nitrogen and carbon isotope analyses. *Earth, Life, and Isotopes*, Kyoto Univ. Press, pp. 339–353 (2010).
38. Ogawa, N.O. et al. Quantification and isotopic measurements of submicrogram scales carbon and nitrogen from extraterrestrial materials through Nano-EA/IRMS. *Lunar and Planetary Science Conference (LPSC)*, #1926 (2020).
39. Suga, H. et al. Sulfur-XANES of intact Ryugu grains and the isolated IOM. *Hayabusa symposium,* # Abstract S32-02 (2022).
40. Hatakeda, K. et al. Homogeneity and heterogeneity in near-infrared FTIR spectra of Ryugu returned samples. *Earth Planets Space* **75**, Article number: 46 (2023).
41. Ehrenfreund, P., Glavin, D.P., Botta, O., Cooper, G. & Bada, J.L. Extraterrestrial amino acids in Orgueil and Ivuna: tracing the parent body of CI type carbonaceous chondrites. *PNAS* **98**, 2138–2141 (2001).
42. Glavin, D.P. et al. The origin and evolution of organic matter in carbonaceous chondrites and links to their parent bodies. *Primitive Meteorites and Asteroids.* Elsevier, pp. 205–271 (2018).
